# Conservative extracorporeal membrane oxygenation treatment in a tracheal injury: a case report

**DOI:** 10.1186/s13019-015-0252-7

**Published:** 2015-04-01

**Authors:** Bong Soo Son, Woo Hyun Cho, Chang Wan Kim, Hyun Min Cho, Seon Hee Kim, Sang Kwon Lee, Do Hyung Kim

**Affiliations:** Departments of Thoracic and Cardiovascular Surgery, Pusan National University Yangsan Hospital, Mulgeum-eup, Yangsan-si, Gyeongnam 626-770 South Korea; Departments of Pulmonary and Critical Care Medicine, Pusan National University Yangsan Hospital, Mulgeum-eup, Yangsan-si, Gyeongnam 626-770 South Korea; Department of Thoracic and Cardiovascular Surgery, Trauma Center of Pusan National University Hospital, Gudeok-ro, Seo-gu, Busan-si South Korea

**Keywords:** Extracorporeal membrane oxygenation, Tracheal injury, Conservative care

## Abstract

In patients with tracheal injuries, conservative treatment is an alternative approach when surgical treatment is difficult. However, the success rate of conservative treatment is low when a ventilator is used constantly because of underlying lung disease, and successful conservative treatment requires the maintenance of as much self-respiration as possible without a ventilator. Here, we report a case of lower tracheal injury in which both surgical and conservative treatments were difficult, but conservative treatment with extracorporeal membrane oxygenation was successful while maintaining self-respiration without a ventilator.

## Background

Immediate surgical repair is the treatment of choice for tracheal injuries. However, most patients undergo emergency surgery while still unstable physiologically, which results in high mortality rates [1,2]. Therefore, conservative treatment is an alternative when surgery is difficult because of the critical condition of the patient or in stable patients when the symptoms are not exacerbated by the tracheal injury [3]. However, successful conservative treatment requires the maintenance of as much self-respiration as possible without a ventilator. Nevertheless, many patients are dependent on ventilators because of underlying respiratory problems, making conservative treatment difficult. Specifically, conservative treatment is impossible in patients with respiratory failure when the tracheal injury is close to the carina. Here, we report a patient with a lower tracheal injury and respiratory failure who was successfully treated conservatively with extracorporeal membrane oxygenation (ECMO), while maintaining self-respiration without a ventilator; in this patient, both surgical and conservative treatments were difficult.

## Case presentation

A 68-year-old female who had a history of diabetes mellitus, chronic renal failure, and coronary heart disease was on ventilator care in the intensive care unit (ICU) because of pulmonary invasive aspergillosis in the right upper lobe and pneumonia. While in the ICU, the tracheostomy site developed sudden, massive bleeding, with resulting airway obstruction. After removing the tracheostomy tube and a large blood clot, an 8–0 intubation tube was inserted into the tracheostomy site. Unfortunately, breathing was not maintained because of the massive bleeding and airway obstruction caused by the hematoma. Her oxygen saturation dropped to 70%. Veno-venous (V-V) ECMO was performed immediately. The right femoral vein was used for venous drainage and the internal jugular vein for venous return. The vessels were cannulated using the Seldinger technique under ultrasound guidance, with a FEM-FLEX2 femoral cannula from Edwards Lifesciences or a Medtronic DLP femoral cannula, respectively. After instituting V-V ECMO, sufficient blood oxygen saturation and a correct carbon dioxide concentration were maintained without ventilation. The endobronchial bleeding was stopped as a consequence of blood clot tamponade, and this was followed by respiratory support without a ventilator, with ECMO only. After confirming that there was no additional bleeding, bronchoscopy was performed to remove the hematoma and determine the site of bleeding.

**Figure 1:**
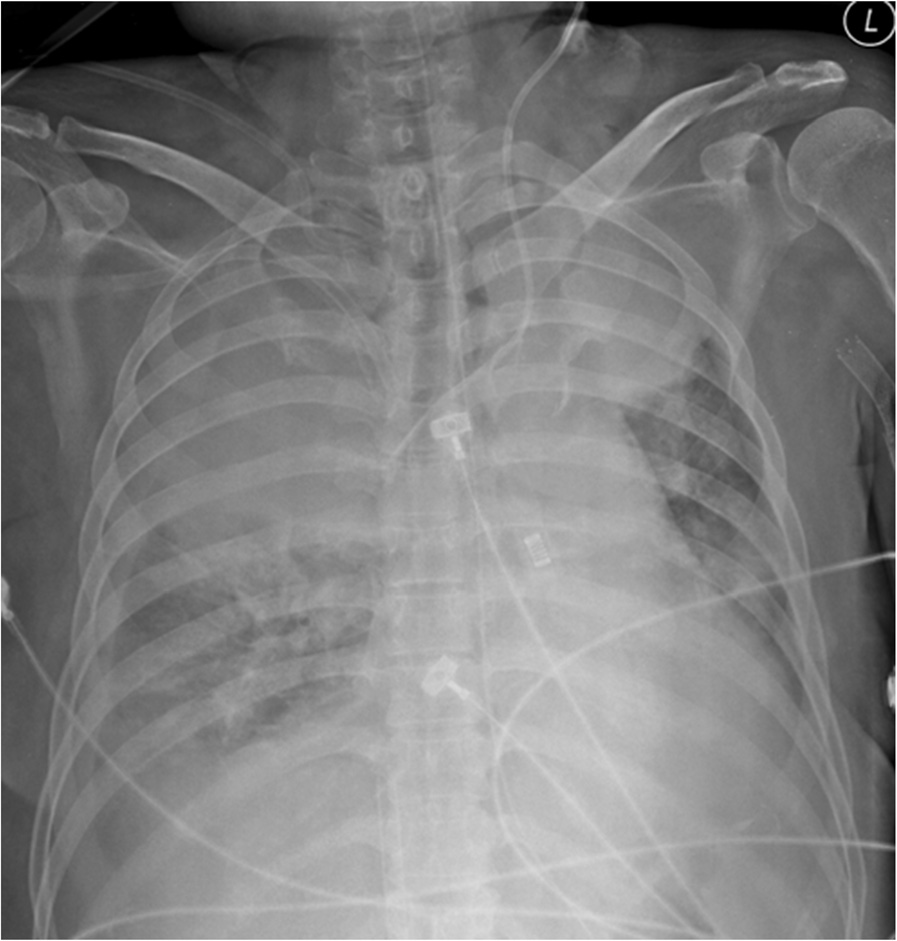
**The chest x-ray after instituting ECMO shows diffuse infiltration in both lungs that developed after massive hemoptysis.**

**Figure 2:**
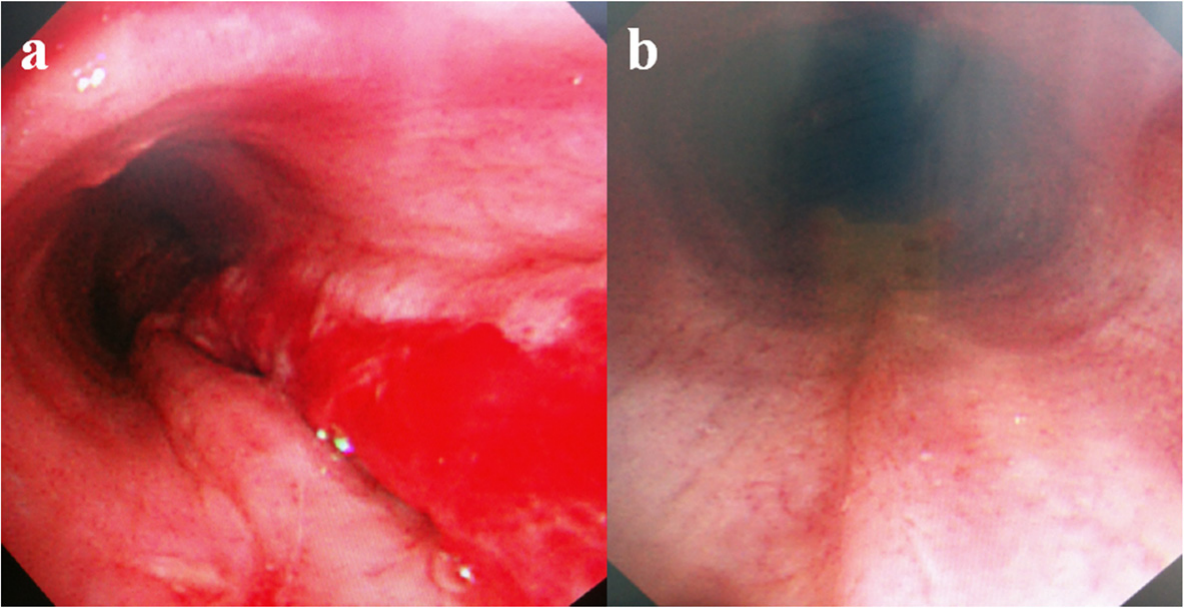
**Bronchoscopic findings. (a)** The bronchoscopic view showing a 5-cm-long full-thickness tear of the membranous trachea extending proximally from the carina. **(b)** Follow-up bronchoscopy on day 40 showing complete healing at the site of the tear in the membranous portion of the trachea.

This revealed an approximately 5-cm tear in the posterior membranous portion 1 cm above the carina. After removing the hematoma, the bleeding site was confirmed to be in the right upper lobe. There was no more bleeding from the site. Emergency surgery was judged to be too difficult to perform because of her underlying disease and acute respiratory distress, which occurred after massive hemoptysis (Figure 1). Maintaining the intubation tube and ventilator care for conservative treatment was also difficult because the injury was close to the carina. Since the patient was able to breathe with ECMO support alone and total airway obstruction, conservative treatment with ECMO seemed appropriate. The patient was kept awake without sedation to allow spontaneous breathing. To maintain the airway without irritating the torn area, a 5–0 tracheostomy tube was kept in place without inflating the balloon, while maintaining an FiO_2_ of 0.5 oxygen supplying 10 L of oxygen per minute through a T-piece. To prevent bleeding, the activated clotting time (ACT) was maintained at about 160 s. The patient’s blood oxygen saturation was set to >95% and the carbon dioxide level to 35–45 mm Hg. We attempted to maintain the optimal oxygen and carbon dioxide levels with the minimum blood flow. ECMO was maintained with self-respiration for 28 days until the patient’s lung condition improved and the injured area had healed. Following ECMO weaning, she was transferred to a general ward 35 days after the tracheal injury. Bronchoscopy 40 days after the injury revealed that the injury had healed (Figure 2a and b).

This was a clinical study and no experimental work. No ethical committee had to be involved. Everything happened in the compliance with the Helsinki Declaration.

## Conclusion

In tracheal injury patients requiring continuous respiratory treatment, the outcome of conservative treatment depends on the location and size of the rupture. If the rupture is in a relatively upper part of the airway, conservative treatment is possible because the respiratory tract can be maintained without additional damage to the rupture site by placing the cuff of the intubation tube below the site of rupture. However, conservative treatment is necessary after selective airway maintenance in the respiratory tract by positioning the intubation tube to one side of the respiratory tract if the rupture site is long and close to the carina. Nevertheless, it is difficult to maintain breathing for weeks with single-lung ventilation alone. In particular, in tracheal injury patients with acute respiratory failure, it is almost impossible to administer conservative treatment for weeks without the development of life-threatening subcutaneous emphysema or pneumomediastinum.

Currently, ECMO is recognized as a means of life support in respiratory failure patients who cannot benefit from conventional ventilator care. ECMO can be used to treat patients with severe pre- or intraoperative tracheal stenosis in order to maintain a stable respiratory status [4,5]. Generally, ECMO is used with a ventilator in patients in respiratory failure. In recent cases, however, ECMO has been used to treat respiratory failure without mechanical ventilation. Treatment with ECMO alone can reduce the complications resulting from long-term ventilator use, as it enables the maintenance of self-respiration [6]. We treated our tracheal injury patient with ECMO monotherapy. The patient maintained stable blood oxygen and carbon dioxide concentrations with ECMO alone, even when ventilation was impossible because of a blood clot; therefore, we decided to treat her conservatively with self-respiration using only ECMO. The patient had an approximately 5-cm-long tracheal injury that healed spontaneously without any noteworthy problems. As long as breathing capability is maintained, ECMO maintenance without complications was possible for the relatively long period of 28 days by maintaining the ACT at approximately 160 s under minimal ECMO flow support and by using a heparin-coated polymethyl pentene oxygenator.

Generally, ECMO support is unnecessary for patients with tracheal injury. Nevertheless, this case demonstrates that conservative treatment with ECMO can be beneficial and increase survival in tracheal injury patients for whom surgery is impossible because of poor general condition, and who require ventilator care for life support, but have difficulty in maintaining an airway.

## Consent

The patient consented to publication of this Case Report and the accompanying images. A copy of the written consent is available for review by the Editor-in-chief of this journal.

## Abbreviation

ECMO: Extracorporeal membranous oxygenation

## References

[1] Miñambres E, Burón J, Ballesteros MA, Llorca J, Muñoz P, González-Castro A (2009). Tracheal rupture after endotracheal intubation: a literature systematic review. Eur J Cardiothorac Surg.

[2] Marty-Ané C-H, Picard E, Jonquet O, Mary H (1995). Membranous tracheal rupture after endotracheal intubation. Ann Thorac Surg.

[3] Gomez-Caro Andres A, Moradiellos Diez FJ, Ausin Herrero P, Kiaz-Hellin Gude V, Larru Cabrero E, de Miguel Porch E (2005). Successful conservative management in iatrogenic tracheobronchial injury. Ann Thorac Surg.

[4] Jeon HK, So YK, Yang JH, Jeong HS (2009). Extracorporeal oxygenation support for curative surgery in a patient with papillary thyroid carcinoma invading the trachea. J Laryngol Otol.

[5] Olsson KM, Simon A, Strueber M, Hadem J, Wiesner O, Gottlieb J (2010). Extracorporeal membrane oxygenation in nonintubated patients as bridge to lung transplantation. Am J Transplantation.

[6] Fuehner T, Kuehn C, Hadem J, Wiesner O, Gottlieb J, Tudorache I (2012). Extracorporeal membrane oxygenation in awake patients as bridge to lung transplantation. Am J Respir Crit Care Med.

